# Protecting P-glycoprotein at the blood–brain barrier from degradation in an Alzheimer’s disease mouse model

**DOI:** 10.1186/s12987-021-00245-4

**Published:** 2021-03-06

**Authors:** Yujie Ding, Yu Zhong, Andrea Baldeshwiler, Erin L. Abner, Björn Bauer, Anika M. S. Hartz

**Affiliations:** 1 Sanders-Brown Center on Aging, University of Kentucky, Lexington, KY 40536 USA; 2 Department of Pharmacy Practice and Pharmaceutical Sciences, College of Pharmacy, University of Minnesota, Duluth, Minnesota 55812 USA; 3 Department of Pharmacology and Nutritional Sciences, University of Kentucky, Lexington, KY 40536 USA; 4 Department of Pharmaceutical Sciences, College of Pharmacy, University of Kentucky, Lexington, KY 40536 USA; 5 Department of Epidemiology, College of Public Health, University of Kentucky, Lexington, KY 40536 USA; 6 University of Kentucky Sanders-Brown Center on Aging, 800 S Limestone, Lexington, KY 40536 USA

**Keywords:** Blood–brain barrier, P-glycoprotein, Alzheimer’s disease, Brain capillaries, Amyloid beta, Ubiquitin-proteasome system

## Abstract

**Background:**

Failure to clear Aβ from the brain is partly responsible for Aβ brain accumulation in Alzheimer’s disease (AD). A critical protein for clearing Aβ across the blood-brain barrier is the efflux transporter P-glycoprotein (P-gp). In AD, P-gp levels are reduced, which contributes to impaired Aβ brain clearance. However, the mechanism responsible for decreased P-gp levels is poorly understood and there are no strategies available to protect P-gp. We previously demonstrated in isolated brain capillaries *ex vivo* that human Aβ40 (hAβ40) triggers P-gp degradation by activating the ubiquitin-proteasome pathway. In this pathway, hAβ40 initiates P-gp ubiquitination, leading to internalization and proteasomal degradation of P-gp, which then results in decreased P-gp protein expression and transport activity levels. Here, we extend this line of research and present results from an *in vivo* study using a transgenic mouse model of AD (human amyloid precursor protein (hAPP)-overexpressing mice; Tg2576).

**Methods:**

In our study, hAPP mice were treated with vehicle, nocodazole (NCZ, microtubule inhibitor to block P-gp internalization), or a combination of NCZ and the P-gp inhibitor cyclosporin A (CSA). We determined P-gp protein expression and transport activity levels in isolated mouse brain capillaries and Aβ levels in plasma and brain tissue.

**Results:**

Treating hAPP mice with 5 mg/kg NCZ for 14 days increased P-gp levels to levels found in WT mice. Consistent with this, P-gp-mediated hAβ42 transport in brain capillaries was increased in NCZ-treated hAPP mice compared to untreated hAPP mice. Importantly, NCZ treatment significantly lowered hAβ40 and hAβ42 brain levels in hAPP mice, whereas hAβ40 and hAβ42 levels in plasma remained unchanged.

**Conclusions:**

These findings provide in vivo evidence that microtubule inhibition maintains P-gp protein expression and transport activity levels, which in turn helps to lower hAβ brain levels in hAPP mice. Thus, protecting P-gp at the blood-brain barrier may provide a novel therapeutic strategy for AD and other Aβ-based pathologies.

## Introduction

One hallmark of Alzheimer’s disease (AD) is the accumulation of neurotoxic amyloid beta (Aβ) in the brain [1]. This accumulation of soluble and insoluble Aβ forms contributes to neurodegeneration and dementia observed in AD [2]. Increasing evidence from recent studies indicates that Aβ brain accumulation is, in part, due to impaired Aβ clearance from the brain across the blood-brain barrier into the blood [3–5]. Results from multiple studies show that the ATP-driven efflux transporter P-glycoprotein (P-gp) transports Aβ, and thus, is involved in clearing Aβ from the brain [6–14]. In this regard, Callaghan et al. used different software docking programs to predict P-gp interactions with Aβ. The obtained models suggest that Aβ40 vertically enters the central cavity of P-gp [7]. Several studies using tissue samples from AD patients have shown that blood-brain barrier P-gp protein expression levels are decreased compared to cognitive normal individuals [15–18]. Recent data show a 53% reduction (*p* < 0.01) in P-gp protein levels in capillaries from AD patient brain samples compared to samples from control individuals [19]. Consistent with decreased P-gp expression levels, results of PET studies indicate compromised P-gp activity levels in AD patients [20, 21]. Thus, existing studies support the conclusion that blood-brain barrier P-gp protein expression and transport activity are decreased in AD. Results from our own studies using a transgenic mouse AD model (human amyloid precursor protein (hAPP)-overexpressing mice; Tg2576) support these observations and suggest a link between high hAβ levels and decreased brain capillary P-gp expression and activity levels in AD [9, 22, 23]. Moreover, we found that hAβ40 causes P-gp degradation in brain capillaries [22, 23]. Specifically, we demonstrated that hAβ40 triggers P-gp degradation through activation of the ubiquitin-proteasome pathway: first, P-gp is ubiquitinated; second, P-gp is internalized; and finally, P-gp is degraded by the proteasome [22, 24]. In a recent study we showed that preventing P-gp ubiquitination by inhibiting the ubiquitin-activating enzyme E1 protects blood-brain barrier P-gp expression and activity levels and lowers hAβ brain levels in young hAPP mice [23]. Here, we extend our work and show that dosing 8-week old hAPP mice with the potent microtubule depolymerization inhibitor nocodazole (NCZ) protects P-gp protein expression and transport activity and lowers hAβ brain levels.

Thus, protecting P-gp from internalization and degradation at the blood-brain barrier could be one strategy to improve Aβ clearance from the brain.

## Materials and methods

### Experimental design and statistical analysis

Sample sizes (i.e., animal numbers, brain capillary sample size, number of liver tissue samples) were based on power analyses of preliminary data and past published work [9, 22, 23, 25]. Sample size and the number of repetitions are reported in the Results section and the corresponding figure legends.

Results are presented as mean ± SEM, or percent change, as indicated. One-way analysis of variance, with factor treatment group, was used to assess mean differences in outcomes. Tukey’s HSD post-hoc test was used to preserve the family-wise Type 1 error rate at 5%. Post hoc tests were not performed when the omnibus F statistic was not significant. Statistical analyses were completed using SAS 9.4^®^ (SAS Institute, Inc.; Cary, NC, USA).

### Animals

The Institutional Animal Care and Use Committee at the University of Minnesota (Protocol #1110A05865, principal investigator; PI: Hartz, AMS) approved all animal experiments, which were conducted in accordance with AAALAC regulations, the Guide of the Care and Use of Laboratory Animals of the NIH, and the US Department of Agriculture Animal Welfare Act.

Male wild type mice (WT; RRID: IMSR_TAC:2789; n = 10) and male transgenic hAPP-overexpressing mice (Tg2576 strain; 129S6.CgTg(APPSWE2576Kha; RRID:IMSR_TAC:2789; n = 45) were acquired from Taconic Farms (Germantown, NY, USA). Mice were received at age 8–12 weeks and were housed individually in an AAALAC-accredited temperature-and-humidity-controlled vivarium (23 °C, 35% relative humidity, 12 h light-dark cycle) and allowed to habituate to their environment for two weeks prior to experiments. Mice had *ad libidum* access to tap water and standard rodent feed (Harlan Teklad Chow 2918, Harlan Laboratories Inc., Indianapolis, NJ, USA). Mice used for experiments were 10–14 weeks old and had a mean body weight of 27.4 ± 1.1 g (mean ± SD) and 27.6 ± 2.7 g (mean ± SD) for WT and hAPP strains, respectively.

### Chemicals

Antibodies against β-actin (ab8226; RRID: AB_306371), human Aβ40 (ab12265; RRID:AB_298985), human Aβ42 (ab12267; RRID:AB_298987), as well as cyclosporin A (CSA; ab120114) were purchased from Abcam (Cambridge, MA, USA). Modified Dulbecco’s phosphate buffered saline (DPBS) with 0.9 mM Ca^2+^ and 0.5 mM Mg^2+^ was purchased from HyClone (Logan, UT, USA). Complete™ protease inhibitor was purchased from Roche (Mannheim, Germany). C219 antibody against P-gp was purchased from ThermoFisher (MA126528; RRID:AB_795165; Waltham, MA, USA). Fluorescein-hAβ_42_ [fluorescein-Aβ_(1− 42)_] was purchased from rPeptide (Bogart, GA, USA). [N-(4-nitrobenzofurazan-7-yl)-D-Lys8]-cyclosporin A (NBD-CSA) was custom-synthesized by R. Wenger (Basel, Switzerland; [26]). PSC833 was a kind gift from Novartis (Basel, Switzerland). Nocodazole, the ALT Assay Kit (MAK052), CelLytic™ M, Ficoll^®^ PM 400, bovine serum albumin and all other chemicals were purchased at the highest grade from Sigma-Aldrich (St. Louis, MO, USA).

### NCZ dosing

Mice were randomly divided into four treatment groups (Group 1: WT mice treated with vehicle, n = 10; Group 2: hAPP mice treated with vehicle, n = 15; Group 3: hAPP mice treated with nocodazole (NCZ), n = 15; and Group 4: hAPP mice treated with cyclosporin A (CSA) and NCZ, n = 15). Mice were treated for a total of 14 days; the dosing regimens for NCZ and CSA are shown in Table 1. The dosing regimen and length of the treatment are based on our previously published study and preliminary experiments using NCZ [23]. Briefly, mice in Groups 1 and 2 were treated daily with vehicle (i.p.) every three days for the entire duration of the experiment. Mice in Groups 3 and 4 were treated with 5 mg/kg of NCZ (i.p.) every 3 days (days 1, 4, 7, 10, and 13). On days when no NCZ was given, mice in Group 3 were treated with vehicle (p.o.) and mice in Group 4 were treated with 25 mg/kg CSA (p.o.).

**Table 1:**
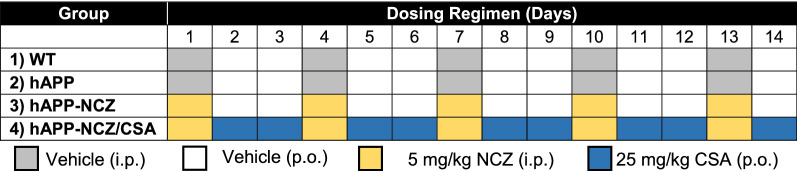
Treatment schedule

### Blood and tissue collection

At the end of the 14-day treatment period, all mice were euthanized by CO_2_ inhalation and decapitated; brain tissue, trunk blood, and liver were collected from each animal. Blood was stored in heparinized tubes and plasma was extracted from blood by centrifugation at 5000*g* for 5 min at 4 °C. Brain and liver tissue were frozen in liquid nitrogen at the time of collection and stored at – 80 °C until further analysis.

### ALT assay

Alanine aminotransferase activity (ALT) in liver tissue lysate was measured using an ALT activity kit (MAK052; Sigma-Millipore, St. Louis, MO, USA) according to the manufacturer’s instructions. Briefly, from each mouse 40 mg of collected liver tissue were homogenized in 300 µl of ALT Assay Buffer using a Power Gen 125 tissue homogenizer (Thermo Fisher Scientific, Hampton, NH, USA). Homogenized liver samples were centrifuged at 15,000*g* for 15 min at 4 °C. Supernatant containing ALT from each liver sample was diluted 1:100 for ALT activity determination. 20 µl of the diluted samples was pipetted in duplicates onto a 96-well microplate. Wells were treated with ALT Master Mix solution. Changes in colorimetric intensity was measured at 570 nm over 30 min (5 min reading intervals, 37 °C) using a Synergy™ H1 Hybrid Multi-Mode Reader (BioTek, Winooski, VT, USA).

The amount of generated pyruvate, a measure of ALT activity, was determined using a standard curve. ALT activity was calculated using Eq. (1).

1 $$\text{A}\text{L}\text{T} \text{A}\text{c}\text{t}\text{i}\text{v}\text{i}\text{t}\text{y}= \frac{(nmol of ALT \times Dilution Factor)}{\left({T}_{final}-{T}_{initial}\right) \times \left(Volume of Sample\right)}$$

ALT activity is reported as nmole/min/ml = milliunit/mL (mU/ml), where one milliunit (mU) of ALT is defined as the amount of enzyme that generates 1.0 nmole of pyruvate per minute at 37 °C.

### Tissue harvest and brain capillary isolation

Brain capillaries were isolated as previously described [22, 23, 27]. Following euthanasia with CO_2_, brains were collected, dissected, and cleaned of meninges. Frontal cortex tissue (~ 10 mg/brain) was collected, snap frozen in liquid nitrogen, and stored at − 80 °C for Aβ analysis. The remaining brain tissue was homogenized in Ca^2+^/Mg^2+^-containing DPBS supplemented with 5 mM D-glucose and 1 mM sodium pyruvate. The brain homogenate was mixed with a 15% Ficoll^→^ PM 400 solution and centrifuged at 5,800 g for 20 min at 4°C to yield capillary-containing pellets. The pellets were separated and resuspended in 1% BSA-DPBS solution and the capillary suspension was filtered through a 300 µm nylon mesh and then passed through a glass bead column (glass bead diameter: 0.45–0.5 mm) with 1% BSA-DPBS. Capillaries were washed off the glass beads with 1% BSA-DPBS. The resulting capillary suspension was centrifuged at 1,500 g for 3 min at 4ºC and the pellet was washed three times with DPBS. Isolated capillaries were used for transport experiments, isolation of capillary crude membranes, or stored for later analysis at − 80 ºC.

### Capillary Crude membrane isolation

Capillary crude membranes were obtained from freshly isolated brain capillaries as described in previous studies [27]. Isolated brain capillaries were homogenized in a cell lysis buffer (CellLytic™ M, Sigma-Aldrich, St. Louis, MO, USA) containing Complete™ protease inhibitor (Sigma-Aldrich, St. Louis, MO, USA). The capillary homogenate was centrifuged at 10,000*g* for 15 min at 4 °C to separate organelles and cellular debris. The supernatant was then centrifuged at 100,000*g* for 90 min at 4°C to obtain a pellet containing brain capillary crude membranes. Crude membranes were resuspended in buffer and stored at − 80 °C.

### Western blotting

Protein expression levels were determined by Western blot analysis as described in previous studies [9, 22]. Protein concentrations of brain capillary crude membrane samples and brain lysate samples were determined with the Bradford assay. The Invitrogen NuPage^®^ Bis-Tris electrophoresis and blotting system was used to perform all Western blots. Following protein transfer, blotting membranes were blocked and incubated overnight with the primary antibody (P-gp: C219, 1 µg/ml; hAβ40: 1 µg/ml; hAβ42: 1 µg/ml; β-Actin: 1 µg/ml). Blotting membranes were washed and incubated for 1 hour with horseradish peroxidase-conjugated ImmunoPure secondary IgG antibody (1:5000, 0.15 µg/ml; Thermo Fisher Scientific, Waltham, MA, USA). Proteins were detected using SuperSignal West Pico Chemiluminescent Substrate (Thermo Fisher Scientific, Waltham, MA, USA). Protein bands were visualized using a Bio-Rad ChemiDoc XRS + gel documentation system. Optical density and digital molecular weight analyses were performed using Image Lab 5.0 software from Bio-Rad Laboratories (RRID:SCR_014210) and a molecular weight marker (RPN800E; GE Healthcare, Chalfont St. Giles, Buckinghamshire, UK). Linear adjustments of contrast and brightness were applied to entire Western blot images. Nonlinear adjustments were not applied.

### P-gp transport activity and p‐gp‐mediated hAβ42 transport

P-gp transport activity levels and P-gp-mediated Aβ transport were determined as previously described [9, 28, 29]. To determine P-gp transport activity, freshly isolated brain capillaries were incubated with 2 µM of the P-gp-specific substrate NBD-cyclosporin A (NBD-CSA) in DPBS for 1 hour. To assess P-gp-mediated hAβ42 transport activity, brain capillaries were incubated with 5 µM fluorescein-hAβ42 in DPBS for 1 hour. Images of 10 capillaries per treatment group were captured by confocal microscopy using the 488 nm line of an argon laser of a Leica TCS SP5 confocal microscope with a 63 × 1.2 NA water immersion objective (Leica Instruments, Wetzlar, Germany). NBD-CSA fluorescence in the capillary lumen was measured in each image using Image J v.1.48v (Wayne Rasband, NIH, USA; RRID:SCR_003070). Specific, luminal NBD-CSA or fluorescein-hAβ42 fluorescence were measured as the difference between total luminal fluorescence and fluorescence in the presence of 5 µM PSC833, a P-gp-specific inhibitor [9, 28, 29].

### **A**β **immunostaining of brain capillaries**

Freshly isolated brain capillaries were immunostained for hAβ40 and hAβ42 using a method described in previous studies [9]. Mouse brain capillaries were fixed with 3% paraformaldehyde/0.25% glutaraldehyde for 30 min at room temperature and subsequently washed with PBS. Fixed capillaries were treated with 0.5% Triton X-100 for 30 min, washed with PBS and blocked with 1% BSA/DPBS for 60 min. Capillaries were incubated overnight using a 1:250 dilution (4 µg/ml) of primary rabbit polyclonal antibody to human Aβ1-40 (hAβ40; ab12265, Abcam, Cambridge, MA, USA; RRID:AB_298985) or primary rabbit polyclonal antibody to human Aβ1-42 (hAβ42; ab12267, Abcam, Cambridge, MA, USA; RRID:AB_298987). Capillaries were washed with 1% BSA/PBS for 60 min and incubated with secondary Alexa-Fluor 488-conjugated goat anti-rabbit IgG (1:1000, 1 µg/ml; Invitrogen, Carlsbad, CA, USA; RRID:AB 2,576,217) for 1 h at 37 °C. Nuclei were counterstained with 1 µg/ml DAPI (MilliporeSigma, Burlington, MA USA;RRID:SCR_014366). Immunofluorescence of hAβ40 and hAβ42 was visualized by confocal microscopy (Leica TCS SP5 confocal microscope, 62 × 1.2 NA water objective, Leica Instruments, Weltzar, Germany). Brain capillary plasma membrane immunofluorescence of hAβ40 and hAβ42 was measured for each capillary with ImageJ software v1.48 as previously described [9, 23]. A 10 × 10 grid was superimposed on each image and fluorescence measurements of capillary membranes were taken between intersecting grid lines. The fluorescence intensity for each capillary was the mean of three measurements per capillary.

### hAβ40 and hAβ42 ELISA

Human Aβ40 and Aβ42 levels in plasma and brain samples were determined by ELISA following the manufacturer’s protocols (KHB3482 (sensitivity: < 6 pg/ml) and KHB3442 (sensitivity: < 10 pg/ml); Invitrogen, Camarillo, CA, USA). Plasma samples were centrifuged at 5000 *g* for 5 min at 4 °C, and then diluted with standard diluent buffer provided in the kit (hAβ40: 1:50 dilution; hAβ42: 1:4 dilution). Brain samples were homogenized in guanidine Tris-HCl buffer (5 M, pH 8) and diluted in DPBS containing 5% BSA and 0.03% Tween-20 (hAβ40: 1:20 dilution; hAβ42: 1:5 dilution). Diluted samples were centrifuged at 16,000*g* for 20 min at 4 °C; the supernatant was analyzed by ELISA. Absorbance (450 nm) was measured using a Synergy™ H1 Hybrid Multi-Mode Reader (BioTek, Winooski, VT, USA). A standard curve was plotted using Gen5™ software v2.07 to determine the concentration of hAβ40 and hAβ42 in plasma and brain samples; values obtained at 450 nm were corrected for background absorbance; four parameter logistic ELISA curve fitting was used.

## Results

We previously reported that hAβ40 triggers degradation of P-gp at the blood-brain barrier [22, 23]. We found that hAβ40 activates the ubiquitin-proteasome system, which leads to internalization and proteasomal degradation of the transporter [22, 24]. Further, we showed that inhibition of intracellular trafficking with microtubule inhibitors blocks proteasomal degradation of P-gp in isolated brain capillaries [22]. The present study extends these findings to an *in vivo* strategy designed to protect P-gp from degradation by blocking transporter internalization.

### Effect of NCZ on P-gp protein expression and transport activity

Microtubule inhibitors, such as nocodazole, have been shown to disrupt intracellular trafficking and internalization of membrane proteins, such as P-gp [22, 30]. In the present study, we used transgenic hAPP mice (Tg2576 model), one of the best characterized AD models that overexpresses human APP which leads to Aβ brain deposits [31]. At 8-weeks of age, hAPP mice have accumulation of human Aβ in the brain and show reduced P-gp protein expression and transport activity at the blood-brain barrier [9, 23]. We dosed 8-week old hAPP mice (n = 15) with the microtubule inhibitor nocodazole (NCZ; 5 mg/kg, i.p.) once every three days for two weeks (Table 1). A second group of hAPP mice received NCZ once every three days and on the two days between NCZ injections, this group of mice also received the P-gp inhibitor cyclosporin A (CSA; 25 mg/kg, p.o.; n = 15). Note that the group of NCZ-CSA-treated hAPP mice served as a control for P-gp transport activity to account for NCZ-treatment effects that depend on P-gp transport activity. Wild Type (WT; n = 10) mice and hAPP control mice (n = 15) received vehicle.

**Fig. 1:**
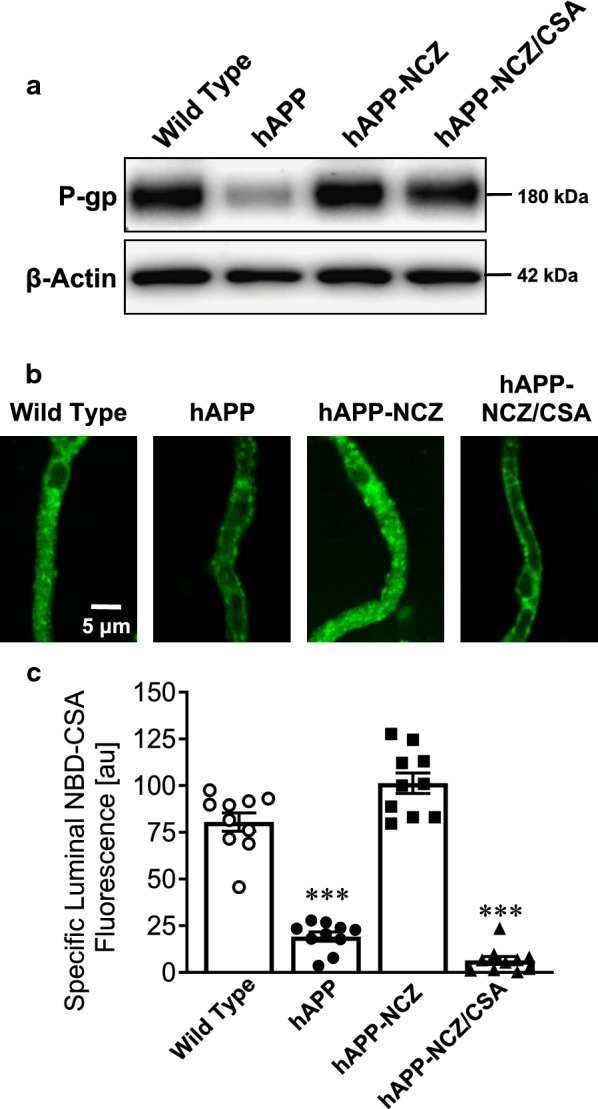
Effect of NCZ on P-gp protein expression and transport activity. **a** Western Blot for P-gp showing bands for brain capillary membranes isolated from vehicle-treated wild-type (WT), vehicle-treated hAPP mice, hAPP mice dosed with 5 mg/kg NCZ and hAPP mice dosed with a combination of NCZ and CSA (5/25 mg/kg). β-Actin was used as protein loading control; pooled tissue (WT: 10 mice; hAPP: 15 mice; hAPP-NCZ: 15 mice, hAPP-NCZ/CSA: 15 mice). Data were normalized to β-Actin. **b** Representative confocal images showing accumulation of P-gp-specific substrate NBD-CSA in isolated brain capillaries from WT, hAPP, hAPP-NCZ and hAPP-NCZ/CSA mice after a 1 hour incubation (steady state; 2 µM NBD-CSA). **c** Data after digital image analysis using ImageJ. Specific NBD-CSA fluorescence is the difference between total luminal NBD-CSA fluorescence and NBD-CSA fluorescence in the presence of the P-gp-specific inhibitor PSC833 (5 µM). Statistics: Data per group are given as mean ± S.E.M. for 10 capillaries from one preparation [pooled tissue: WT (10 mice), hAPP (15 mice), hAPP-NCZ (15 mice), hAPP-NCZ/CSA (15 mice)]. Shown are arbitrary fluorescence units (scale 0–255). ***, significantly lower than control, *p* < 0.001

We found that after 14 days of treatment, P-gp protein expression levels in brain capillary membranes from NCZ-treated hAPP mice were comparable to those observed in WT mice (Fig. 1a). The same effect was observed in NCZ-CSA-treated mice. In contrast, vehicle-treated hAPP mice showed significantly decreased P-gp protein expression levels. Western blots were quantified by optical density analysis and normalized to β-actin (Table 2). Optical density measurements of P-gp protein expression levels (n = 3, normalized to β-actin, SEM, *p*-value) are reported as percentage relative to WT control mice: hAPP: 51 ± 3% (Δ=-49%; *p* = 0.0093; Tukey’s HSD *post hoc* test); hAPP-NCZ: 102 ± 7% (Δ = 2%; *p* = 1.00; Tukey’s HSD *post hoc* test), and hAPP-NCZ/CSA: 91 ± 6% (Δ=-9%; *p* = 0.83; Tukey’s HSD *post hoc* test).

**Table 2:** Western blot analysis of P-gp protein expression levels in brain capillaries isolated from WT and hAPP mice (Fig. 1a)

Protein	Wild Type	hAPP	hAPP-NCZ	hAPP-NCZ/CSA
P-gp	100 ± 13	51 ± 3*	102 ± 7	91 ± 6

Data were obtained from optical density measurements and were normalized to β-actin levels; values are given as %control ± SEM (n = 3)

*Indicates statistical significance determined at the 0.05 level for each endpoint compared to control using Dunnett’s many-to-one *t*-test

We also determined P-gp transport activity levels by live-cell imaging of isolated brain capillaries according to a previously established protocol [9, 22, 25, 28]. Briefly, freshly isolated brain capillaries are transferred onto a glass cover slip and incubated for 1 hour with the fluorescent P-gp substrate NBD-cyclosporin A (NBD-CSA, 2 µM). Brain capillaries are then imaged using confocal microscopy and fluorescence of NBD-CSA accumulation in the capillary lumen is measured by quantitative image analysis using ImageJ. Changes in luminal NBD-CSA fluorescence compared to WT control indicate changes in P-gp transport activity. Thus, luminal NBD-CSA fluorescence is an indirect measure of P-gp transport activity. Figure 1b shows representative confocal images of brain capillaries isolated from WT mice, hAPP mice, NCZ-treated hAPP mice, and hAPP mice treated with the combination of NCZ-CSA after a one-hour exposure to 2 µM NBD-CSA. Image analysis shows that specific luminal NBD-CSA fluorescence levels in the lumens of brain capillaries from hAPP mice were decreased by 76% (*p* < 0.0001; Tukey’s HSD *post hoc* test) compared to levels in capillaries from WT mice (Fig. 1c). Luminal NBD-CSA fluorescence levels in brain capillaries from NCZ-treated hAPP mice were increased by 26% relative to levels measured in capillaries from WT mice (*p* = 0.03; Tukey’s HSD *post hoc* test). Compared to WT mice, luminal NBD-CSA fluorescence levels in brain capillaries from NCZ-treated hAPP mice that received CSA were reduced by 92%. This decrease in luminal NBC-CSA fluorescence is comparable to that found in vehicle-treated hAPP mice (*p* < 0.0001; Tukey’s HSD *post hoc* test). Thus, we found decreased luminal NBD-CSA fluorescence in brain capillaries isolated from untreated hAPP mice and NCZ-CSA-treated hAPP mice, whereas luminal NBD-CSA fluorescence levels in brain capillaries from NCZ-treated hAPP mice were similar to those observed in capillaries from WT mice. Note that we previously published a control experiment showing that nocodazole alone has no effect on P-gp protein expression and transport activity in isolated brain capillaries [22]. Thus, it is unlikely that the observed effects are due to nocodazole-mediated induction of P-gp protein expression.

In summary, the findings described in Fig. 1 show that treating hAPP mice with NCZ for two weeks restored P-gp protein expression and transport activity levels in brain capillaries. The data presented here together with our previously published work suggest that NCZ likely prevents P-gp internalization and degradation [22, 23].

### Effect of NCZ on p‐gp‐mediated Aβ transport in hAPP mice

**Fig. 2:**
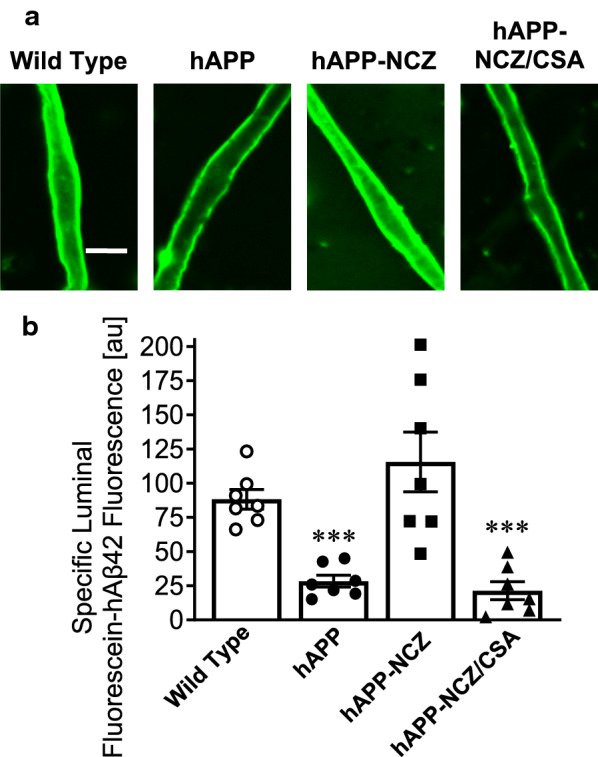
Effect of NCZ on P-gp-Mediated hAβ42 transport. **a** Representative confocal images of isolated brain capillaries showing accumulation of fluorescein-hAβ42 in capillary lumens after a 1 hour incubation (steady state; 5 µM fluorescein-hAβ42). **b** Data after digital image analysis using ImageJ. Specific fluorescein-hAβ42 fluorescence is the difference between total luminal fluorescein-hAβ42 fluorescence and fluorescein-hAβ42 fluorescence in the presence of the P-gp-specific inhibitor PSC833 (5 µM). Statistics: Data per group are given as mean ± S.E.M. for 10 capillaries from one preparation (pooled tissue: WT (10 mice), hAPP (15 mice), hAPP-NCZ (15 mice), hAPP-NCZ/CSA (15 mice)). Shown are arbitrary fluorescence units (scale 0–255). ***, significantly lower than control, *p* < 0.001

Next, we assessed P-gp-mediated transport of fluorescent labeled hAβ42 in isolated brain capillaries from mice in all four treatment groups. In these experiments, isolated brain capillaries were incubated to steady state with fluorescein-hAβ42 (FL-hAβ42; 5 µM) for 1 hour before we imaged the capillaries with a confocal microscope. FL-hAβ42 fluorescence in capillary lumens was analyzed by quantitative image analysis [9, 22, 25, 28]. Figure 2a shows representative confocal images of isolated brain capillaries incubated to steady state with 5 µM FL-hAβ42. These images show reduced luminal FL-hAβ42 fluorescence in capillaries from vehicle-treated hAPP mice and in hAPP mice treated with NCZ-CSA compared to WT and NCZ-treated hAPP mice. Note that CSA is a competitive P-gp inhibitor, and therefore, P-gp-mediated FL-hAβ42 transport is reduced in isolated capillaries from hAPP mice that received the combination NCZ-CSA. Image analysis shows that in capillaries from hAPP mice, luminal FL-hAβ42 fluorescence was decreased by 68% (*p* = 0.0023; Tukey’s HSD *post hoc* test) compared to capillaries from WT mice (Fig. 2b). In brain capillaries from NCZ-CSA-treated hAPP mice, luminal FL-hAβ42 fluorescence is significantly decreased by 68% (*p* = 0.0007; Tukey’s HSD *post hoc* test) compared to that in capillaries from WT control mice. In contrast, luminal FL-hAβ42 fluorescence in capillaries from NCZ-treated hAPP mice was comparable to levels found in WT control mice (Δ = 27%; *p* = 0.27; Tukey’s HSD *post hoc* test).

Together, the data in Figs. 1 and 2 suggest that treatment of hAPP mice with NCZ protects P-gp protein expression and transport activity at the blood-brain barrier.

### Effect of NCZ treatment on ALT levels

**Fig. 3:**
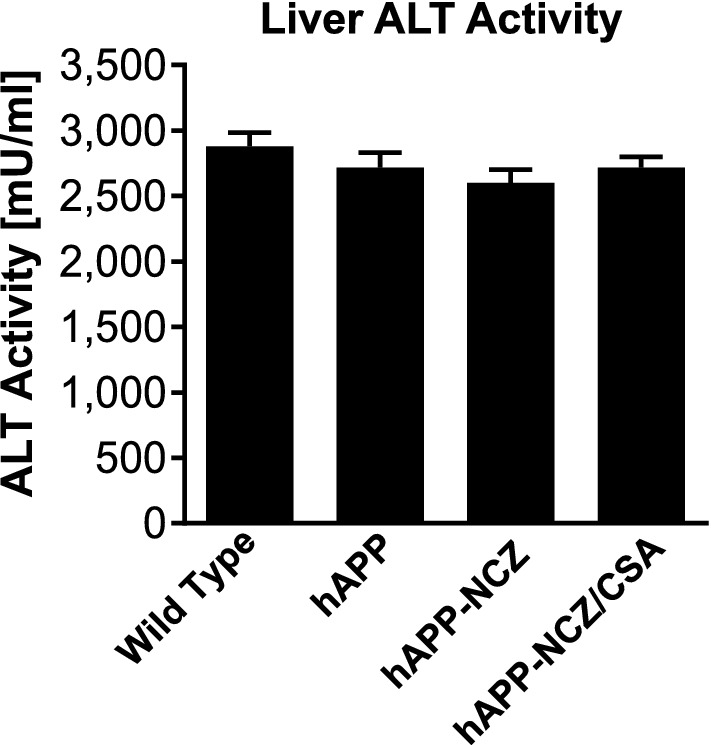
NCZ shows no effect on ALT activity levels. ALT activity levels (mU/ml) in liver samples from WT, hAPP, hAPP-NCZ and hAPP-NCZ/CSA mice. Data are given for each animal: WT (n = 10), hAPP (n = 15), hAPP-NCZ (n = 15), hAPP-NCZ/CSA (n = 15). Statistics: ANOVA; Data between groups are not significantly different

During the two-week treatment with NCZ, animals were continuously monitored by daily health checks, and we observed no NCZ-induced visible adverse effects. We also determined alanine-aminotransferase (ALT) activity levels in liver tissue samples from treated and untreated animals after the 2-week dosing period to determine potential NCZ-induced liver toxicity. Increased ALT activity levels can be an indicator of liver damage and toxicity and are generally used in the clinic to monitor overall health, in particular to detect drug-induced liver toxicity [32]. ALT activity levels from all treatment groups ranged between 2,599 and 2,881 mU/ml; there was no significant difference between any of the groups (F_3,51_=1.1, *p* = 0.36; ANOVA; Fig. 3).

These data show that the 2-week NCZ treatment did not affect ALT activity, which suggests that NCZ did not induce liver damage in the animals during the treatment phase. More studies are necessary to fully evaluate the safety of NCZ, especially with regard to a long-term treatment regimen.

### Effect of NCZ treatment on Aβ levels in hAPP mice

Next we evaluated the effect of NCZ treatment on hAβ40 and hAβ42 levels in plasma, brain capillary membranes, and brain tissue.

**Fig. 4:**
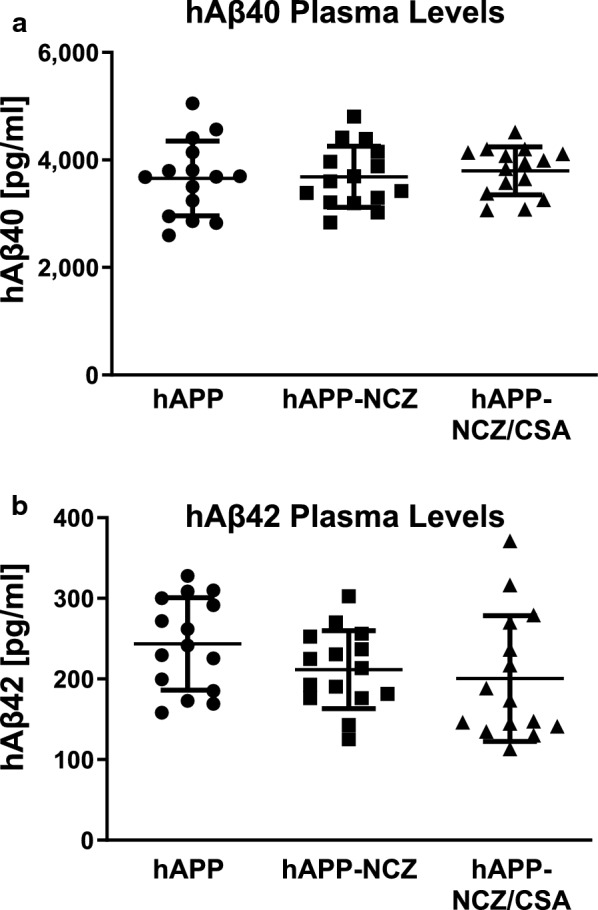
NCZ treatment has no effect on hAβ40 and hAβ42 plasma levels in hAPP mice. **a** hAβ40 levels (pg/ml) and **b** hAβ42 levels (pg/ml) in plasma samples from WT, hAPP, hAPP-NCZ and hAPP-NCZ/CSA mice. Data are given for each animal: WT (n = 10), hAPP (n = 15), hAPP-NCZ (n = 15), hAPP-NCZ/CSA (n = 15). Statistics: ANOVA; Data between groups are not significantly different

We used ELISA to determine plasma hAβ40 and hAβ42 protein levels (Fig. 4a, b). hAβ40 levels were in the range 3,656–3,797 pg/ml with no significant difference between groups (F_2,42_, *p* = 0.98; ANOVA). For hAβ42, we found levels ranging from 200 to 244 pg/ml, also with no significant difference between groups (F_2,42_=0.13, *p* = 0.88; ANOVA). This finding is consistent with rapid Aβ clearance from blood by the liver and the kidney [33]. Note that samples from WT mice were not included in the analysis since WT mice do not express human Aβ.

**Fig. 5:**
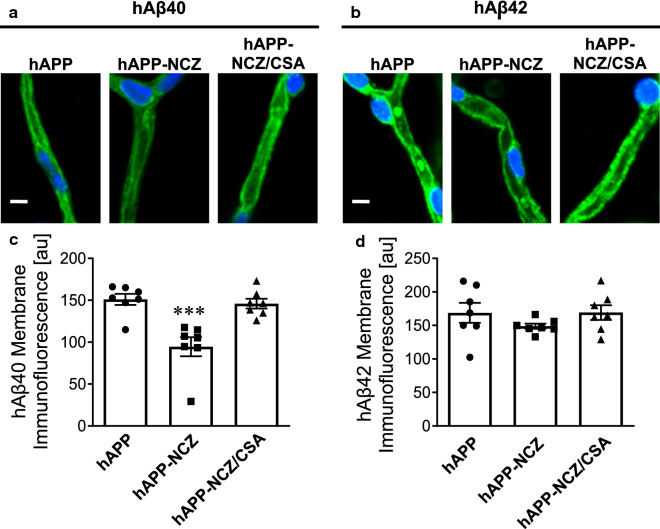
NCZ lowers Aβ levels in brain capillaries. Representative confocal images of isolated brain capillaries immunostained for **a** hAβ40 and **b** hAβ42. Data after digital image analysis of membrane-associated **c** hAβ40- and (D) hAβ42-immunofluorescence using ImageJ. Statistics: Data per group are given as mean ± S.E.M. for 10 capillaries from one preparation [pooled tissue: WT (n = 10), hAPP (n = 15), hAPP-NCZ (n = 15), hAPP-NCZ/CSA (n = 15)]. Shown are arbitrary fluorescence units (scale 0–255). For hAβ40 *p* < 0.001; ***, significantly lower than control. For hAβ42, data between groups are not significantly different

One characteristic of AD is accumulation of Aβ in blood vessel walls [27, 34]. We therefore determined capillary-associated hAβ levels with immunohistochemistry. Freshly isolated brain capillaries were immunostained for hAβ40 and hAβ42, imaged with a confocal microscope, followed by image analysis of capillary membranes. Brain capillaries isolated from treated and untreated hAPP mice stained positive for both hAβ40 and hAβ42 (Fig. 5a, b). Image analysis revealed that NCZ-treatment lowered capillary-associated hAβ40 by 37% (*p* = 0.0004; Tukey’s HSD *post hoc* test) compared to capillaries from vehicle-treated hAPP mice. For hAβ42, no difference was found between treatment groups (*p* = 0.91; Tukey’s HSD *post hoc* test).

**Fig. 6:**
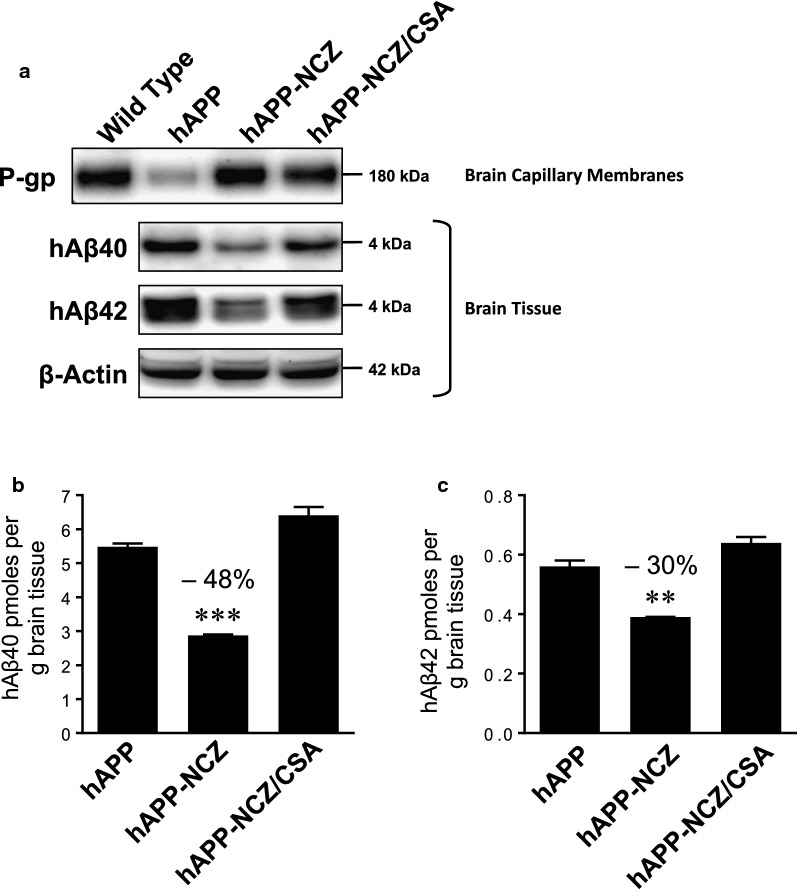
NCZ lowers Aβ brain levels. **a** Western Blot showing hAβ40 and hAβ42 protein expression levels in brain tissue samples from vehicle-treated WT, vehicle-treated hAPP mice, hAPP mice dosed with 5 mg/kg NCZ and hAPP mice dosed with a combination of NCZ and CSA (5/25 mg/kg). β-Actin was used as protein loading control. **b** hAβ40 and **c** hAβ42 levels (pg/ml) in brain tissue samples from hAPP, hAPP-NCZ and hAPP-NCZ/CSA mice determined by ELISA. Statistics: ***, significantly lower than control, *p* < 0.001; **, significantly lower than control, *p* < 0.01

Finally, we analyzed hAβ40 and hAβ42 brain levels by Western blotting and ELISA. Western blotting showed reduced hAβ40 and hAβ42 protein levels in brain tissue samples from NCZ-treated hAPP mice compared to untreated hAPP mice (Fig. 6a). In contrast, hAβ protein levels in brain tissue from NCZ-treated mice that also received CSA to control for P-gp transport activity appeared similar to those detected in brain tissue from vehicle-treated hAPP mice. Using ELISA, we found that in NCZ-treated hAPP mice hAβ40 brain levels were reduced by 48% (*p* < 0.0001; Tukey’s HSD *post hoc* test) and hAβ42 brain levels were reduced by 30% (*p =* 0.0008; Tukey’s HSD *post hoc* test) compared to vehicle-treated hAPP mice (Fig. 6b, c).

**Fig. 7:**
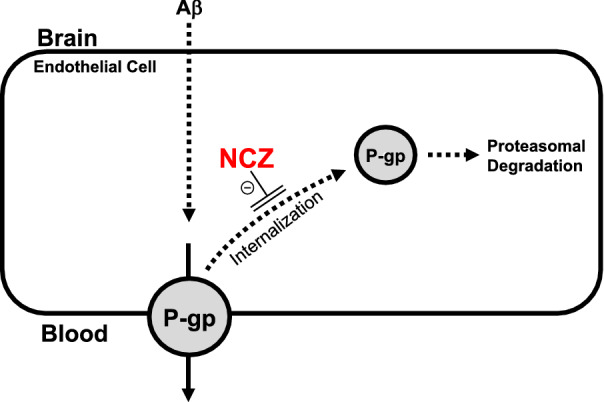
Proposed mechanism. Based on our data presented here and our previously published data [22, 23], we propose that blocking Aβ-induced internalization protects P-gp from proteasomal degradation, which preserves P-gp protein expression and transport activity and helps to reduce Aβ brain levels in hAPP mice

In summary, our data presented here together with our previously publish work support our proposed mechanism that blocking P-gp internalization with NCZ protects P-gp from proteasomal degradation, which helps reduce Aβ brain load in hAPP mice (Fig. 7).

## Discussion

We previously showed that hAβ40 triggers (1) ubiquitination, (2) internalization, and (3) proteasomal degradation of blood-brain barrier P-gp, which results in decreased P-gp protein expression and transport function [22, 24]. We further demonstrated that preventing P-gp ubiquitination preserves P-gp protein expression and transport activity at the blood-brain barrier, which resulted in lower Aβ brain levels in vivo [23]. Our present study builds on this existing work and extends these findings by targeting Aβ-driven P-gp internalization in vivo.

For the present study, we treated hAPP mice with the microtubule inhibitor, nocodazole (NCZ), and evaluated the effects of NCZ on P-gp protein expression and transport activity and hAβ burden. We found that isolated brain capillaries from hAPP mice treated with NCZ had P-gp expression and transport activity levels comparable to those from WT control mice (Figs. 1 and 2). No adverse effects were noticed during nocodazole treatment, accordingly, ALT activity levels in liver samples were similar across all groups. The absence of adverse effects is likely due to the relatively short treatment phase of 2 weeks. (Fig. 3). No difference in hAβ40 and hAβ42 levels was detected in plasma samples from treated and un-treated hAPP mice (Fig. 4). We found that NCZ treatment decreased membrane-associated hAβ40 levels in hAPP mice (Fig. 5) and show that hAβ40 and hAβ42 levels were significantly lower in brain samples from NCZ-treated hAPP mice compared to those in untreated hAPP mice and hAPP-NCZ/CSA control mice (Fig. 6). Based on these data we conclude that NCZ treatment protects P-gp protein expression and transport activity levels at the blood-brain barrier and results in reduced Aβ levels (Fig. 7). This effect is likely due to the inhibition of P-gp internalization and degradation by NCZ. In the following sections we discuss our findings in the context of the existing literature.

One mechanism that accounts for changes in P-gp-mediated transport is trafficking between vesicular compartments and the plasma membrane. Arias and colleagues were the first to report membrane trafficking of several ABC transporters, including P-gp, bile salt export pump (BSEP), and Mrp2 at the bile canalicular membrane of hepatocytes [35, 36]. These authors showed cycling of P-gp between intracellular pools and the plasma membrane by demonstrating insertion of P-gp into the canalicular membrane in response to cyclic AMP (cAMP) and taurocholate. This effect was independent from protein biosynthesis. In addition, the microtubule disruptor, colchicine, and the PI3 kinase inhibitor, wortmannin, inhibited membrane insertion of P-gp. On the other hand, it was also demonstrated that cholestasis caused retrieval of P-gp from the canalicular membrane into intracellular vesicles [35, 37, 38]. Sai et al. visualized P-gp trafficking between the pericanalicular region and the bile canalicular membrane in a hepatic cell line expressing GFP-tagged P-gp [36]. These researchers also demonstrated an involvement of microtubules in P-gp membrane trafficking. Their results show that in hepatocytes, vesicular trafficking of P-gp depends on microtubules and modulates transport activity at the canalicular membrane. Recent studies indicate that P-gp trafficking is also an important mechanism to regulate P-gp transport activity at the blood-brain barrier. Hawkins and colleagues found that the vascular endothelial growth factor (VEGF) triggers removal of P-gp from the luminal membrane resulting in decreased P-gp transport activity in brain capillaries [39]. This study provided first evidence that loss of P-gp transport activity can be due to P-gp internalization shifting the transporter into an intracellular compartment where it no longer functions as an efflux transporter. The effect of VEGF was blocked by nocodazole, whereas proteasome inhibition had no effect. In a series of elegant studies, McCaffrey, Tome and colleagues discovered that peripheral inflammatory pain leads to caveolin-1-mediated trafficking of P-gp at the blood-brain barrier [40–42]. Caveolin-1 is a scaffolding protein that has been shown to interact with P-gp [43]. In the case of peripheral pain, P-gp levels increased at the luminal membrane of the brain capillary endothelium. In an *in vitro* study, Noack et al. found that drugs such as mitomycin C trigger P-gp trafficking in the hCMEC/D3 cell line [44]. Redistribution of P-gp from intracellular pools to the cell membrane occurred within 2 hours of incubation with mitomycin C. Little is known about signaling steps through which P-gp internalization and/or trafficking of the transporter to the membrane occurs at the blood-brain barrier. Work in liver and cancer cells showed that Src kinase interacts with P-gp and that this interaction is likely responsible for P-gp membrane localization [45]. Several studies found that Pim-1 kinase phosphorylates P-gp, which seems to be necessary for plasma membrane localization of the transporter [46–49]. However, the role Pim-1 plays in P-gp internalization and trafficking still needs to be determined in more detail. We recently observed that nanomolar concentrations of hAβ40 trigger P-gp degradation through the ubiquitin-proteasome system [22, 24]. Inhibiting protein ubiquitination, protein trafficking, and the proteasome prevented hAβ40-mediated decrease of P-gp protein expression and transport activity. In a subsequent study we showed that inhibiting the ubiquitin-activating enzyme E1 with PYR41 prevented P-gp ubiquitination *in vivo* prevented P-gp degradation and lowered Aβ brain levels [23]. The present study expands this work by treating hAPP mice with the microtubule inhibitor nocodazole in vivo and demonstrates that P-gp expression and activity can be protected with NCZ. In both studies, we used prefrontal cortex for Aβ quantification since this brain region is highly affected in AD and shows early signs of Aβ deposition, while the remaining part of the cerebral cortex was used to isolate brain capillaries. Comparing the data from our previous study with PYR41 and the data from the present study with nocodazole, we found no difference between the effects of PYR41 and nocodazole on protecting P-gp from degradation and decreasing Aβ brain levels (Aβ40: NCZ 48% vs. PYR41 53% and Aβ42: NCZ 30% vs. PYR41 33%).

Drug combinations are often applied to treat complex diseases and synergistic effects among different drugs hold the promise to achieve larger effects with lower doses and reduced side effect profiles [50]. Drugs acting on the same signaling pathway by targeting different signaling molecules are more likely to produce synergistic effects. Thus, based on our proposed signaling pathway (Fig. 7), inhibiting both ubiquitination and internalization at the same time has the potential for synergistic effects on preserving P-gp and lowering Aβ brain levels. Clearly, future studies are needed to demonstrate such potentially synergistic effects.

## Conclusions

Based on our previously published work and the data presented here, we conclude that inhibiting P-gp internalization with a microtubule inhibitor could be used to protect P-gp from degradation which could help lower Aβ brain levels in AD [22, 23]. Therefore, modulating P-gp trafficking could be a potential new therapeutic target to help protect P-gp at the blood-brain barrier in AD. Currently, microtubule inhibitors are approved for solid and hematologic cancers and include agents such as taxanes, vinca alkaloids, and epothilones. Several drugs including vincristine and colcemid are similar to nocodazole in that they interfere with microtubule polymerization [51]. Thus, there is the possibility of therapeutically targeting P-gp trafficking in AD. However, neuronal activity depends to a large extent on vesicular trafficking, and thus, a microtubule inhibitor would be needed that is suitable and safe for the use in CNS disorders including AD. Clearly, more work is needed to evaluate the potential therapeutic benefit of blocking P-gp internalization in AD.

## Data Availability

The datasets used and/or analyzed during the current study are available from the corresponding author on reasonable request.

## References

[1] Gravina SA, Ho L, Eckman CB, Long KE, Otvos L, Younkin LH (1995). Amyloid beta protein (A beta) in Alzheimer’s disease brain. Biochemical and immunocytochemical analysis with antibodies specific for forms ending at A beta 40 or A beta 42(43). J Biol Chem.

[2] Hardy J, Selkoe DJ (2002). The amyloid hypothesis of Alzheimer’s disease: progress and problems on the road to therapeutics. Science.

[3] Shibata M, Yamada S, Kumar SR, Calero M, Bading J, Frangione B (2000). Clearance of Alzheimer’s amyloid-ss(1–40) peptide from brain by LDL receptor-related protein-1 at the blood-brain barrier. J Clin Invest.

[4] Frangione Zlokovic B (2003). Transport-clearance hypothesis for Alzheimer’s disease and potential therapeutic implications. Landes Biosci.

[5] Zlokovic BV, Deane R, Sallstrom J, Chow N, Miano JM (2005). Neurovascular pathways and Alzheimer amyloid beta-peptide. Brain Pathol..

[6] Bruckmann S, Brenn A, Grube M, Niedrig K, Holtfreter S, von Halbach B (2017). Lack of P-glycoprotein Results in Impairment of Removal of Beta-Amyloid and Increased Intraparenchymal Cerebral Amyloid Angiopathy after Active Immunization in a Transgenic Mouse Model of Alzheimer’s Disease. Curr Alzheimer Res.

[7] Callaghan R, Gelissen IC, George AM, Hartz AMS (2020). Mamma Mia, P-glycoprotein binds again. FEBS Lett..

[8] Cirrito JR, Deane R, Fagan AM, Spinner ML, Parsadanian M, Finn MB (2005). P-glycoprotein deficiency at the blood-brain barrier increases amyloid-beta deposition in an Alzheimer disease mouse model. J Clin Invest.

[9] Hartz AM, Miller DS, Bauer B (2010). Restoring blood-brain barrier P-glycoprotein reduces brain amyloid-beta in a mouse model of Alzheimer’s disease. Mol Pharmacol.

[10] Kuhnke D, Jedlitschky G, Grube M, Krohn M, Jucker M, Mosyagin I (2007). MDR1-P-Glycoprotein (ABCB1) Mediates Transport of Alzheimer’s amyloid-beta peptides–implications for the mechanisms of Abeta clearance at the blood-brain barrier. Brain Pathol.

[11] Lam FC, Liu R, Lu P, Shapiro AB, Renoir JM, Sharom FJ (2001). beta-Amyloid efflux mediated by p-glycoprotein. J Neurochem.

[12] McCormick JA, Chen L, Vogel G, Wise PDJG (2020). Transport of Alzheimer’s Associated Amyloid-beta Catalyzed by P-glycoprotein. bioRxiv.

[13] Wang W, Bodles-Brakhop AM, Barger SW (2016). A Role for P-Glycoprotein in Clearance of Alzheimer Amyloid beta -Peptide from the Brain. Curr Alzheimer Res.

[14] Yuede CM, Lee H, Restivo JL, Davis TA, Hettinger JC, Wallace CE (2016). Rapid in vivo measurement of beta-amyloid reveals biphasic clearance kinetics in an Alzheimer’s mouse model. J Exp Med.

[15] Carrano A, Snkhchyan H, Kooij G, van der Pol S, van Horssen J, Veerhuis R (2014). ATP-binding cassette transporters P-glycoprotein and breast cancer related protein are reduced in capillary cerebral amyloid angiopathy. Neurobiol Aging.

[16] Chiu C, Miller MC, Monahan R, Osgood DP, Stopa EG, Silverberg GD (2015). P-glycoprotein expression and amyloid accumulation in human aging and Alzheimer’s disease: preliminary observations. Neurobiol Aging.

[17] Jeynes B, Provias J (2011). An investigation into the role of P-glycoprotein in Alzheimer’s disease lesion pathogenesis. Neurosci Lett.

[18] Wijesuriya HC, Bullock JY, Faull RL, Hladky SB, Barrand MA (2010). ABC efflux transporters in brain vasculature of Alzheimer’s subjects. Brain Res.

[19] Kannan P, Schain M, Kretzschmar WW, Weidner L, Mitsios N, Gulyas B (2017). An automated method measures variability in P-glycoprotein and ABCG2 densities across brain regions and brain matter. J Cereb Blood Flow Metab.

[20] Deo AK, Borson S, Link JM, Domino K, Eary JF, Ke B (2014). Activity of P-Glycoprotein, a beta-Amyloid Transporter at the Blood-Brain Barrier, Is Compromised in Patients with Mild Alzheimer Disease. J Nucl Med.

[21] van Assema DM, Lubberink M, Boellaard R, Schuit RC, Windhorst AD, Scheltens P (2012). P-glycoprotein function at the blood-brain barrier: effects of age and gender. Mol Imaging Biol.

[22] Hartz AM, Zhong Y, Wolf A, LeVine H, Miller DS, Bauer B (2016). Abeta40 Reduces P-Glycoprotein at the Blood-Brain Barrier through the Ubiquitin-Proteasome Pathway. J Neurosci.

[23] Hartz AMS, Zhong Y, Shen AN, Abner EL, Bauer B (2018). Preventing P-gp Ubiquitination Lowers Abeta Brain Levels in an Alzheimer’s Disease Mouse Model. Front Aging Neurosci.

[24] Akkaya BG, Zolnerciks JK, Ritchie TK, Bauer B, Hartz AM, Sullivan JA (2015). The multidrug resistance pump ABCB1 is a substrate for the ubiquitin ligase NEDD4-1. Mol Membr Biol.

[25] Hartz AM, Pekcec A, Soldner EL, Zhong Y, Schlichtiger J, Bauer B (2017). P-gp Protein Expression and Transport Activity in Rodent Seizure Models and Human Epilepsy. Mol Pharm.

[26] Wenger RM (1986). Cyclosporine and analogues–isolation and synthesis–mechanism of action and structural requirements for pharmacological activity. Fortschr Chem Org Naturst.

[27] Hartz AM, Bauer B, Soldner EL, Wolf A, Boy S, Backhaus R (2012). Amyloid-beta contributes to blood-brain barrier leakage in transgenic human amyloid precursor protein mice and in humans with cerebral amyloid angiopathy. Stroke.

[28] Hartz AM, Bauer B, Block ML, Hong JS, Miller DS (2008). Diesel exhaust particles induce oxidative stress, proinflammatory signaling, and P-glycoprotein up-regulation at the blood-brain barrier. FASEB J.

[29] Hartz AM, Bauer B, Fricker G, Miller DS (2004). Rapid regulation of P-glycoprotein at the blood–brain barrier by endothelin-1. Mol Pharmacol.

[30] Kim NH, Chung KS, Day BN (1997). The distribution and requirements of microtubules and microfilaments during fertilization and parthenogenesis in pig oocytes. J Reprod Fertil.

[31] Hsiao K, Chapman P, Nilsen S, Eckman C, Harigaya Y, Younkin S (1996). Correlative memory deficits, Abeta elevation, and amyloid plaques in transgenic mice. Science.

[32] Liu Z, Que S, Xu J, Peng T (2014). Alanine aminotransferase-old biomarker and new concept: a review. Int J Med Sci.

[33] Ghiso J, Shayo M, Calero M, Ng D, Tomidokoro Y, Gandy S (2004). Systemic catabolism of Alzheimer’s Abeta40 and Abeta42. J Biol Chem.

[34] Love S, Miners S, Palmer J, Chalmers K, Kehoe P (2009). Insights into the pathogenesis and pathogenicity of cerebral amyloid angiopathy. Front Biosci (Landmark Ed).

[35] Kipp H, Arias IM (2002). Trafficking of canalicular ABC transporters in hepatocytes. Annu Rev Physiol.

[36] Sai Y, Nies AT, Arias IM (1999). Bile acid secretion and direct targeting of mdr1-green fluorescent protein from Golgi to the canalicular membrane in polarized WIF-B cells. J Cell Sci.

[37] Gatmaitan ZC, Nies AT, Arias IM (1997). Regulation and translocation of ATP-dependent apical membrane proteins in rat liver. Am J Physiol.

[38] Kipp H, Pichetshote N, Arias IM (2001). Transporters on demand: intrahepatic pools of canalicular ATP binding cassette transporters in rat liver. J Biol Chem.

[39] Hawkins BT, Rigor RR, Miller DS (2010). Rapid loss of blood–brain barrier P-glycoprotein activity through transporter internalization demonstrated using a novel in situ proteolysis protection assay. J Cereb Blood Flow Metab.

[40] McCaffrey G, Staatz WD, Sanchez-Covarrubias L, Finch JD, Demarco K, Laracuente ML (2012). P-glycoprotein trafficking at the blood-brain barrier altered by peripheral inflammatory hyperalgesia. J Neurochem.

[41] Tome ME, Herndon JM, Schaefer CP, Jacobs LM, Zhang Y, Jarvis CK (2016). P-glycoprotein traffics from the nucleus to the plasma membrane in rat brain endothelium during inflammatory pain. J Cereb Blood Flow Metab.

[42] Tome ME, Jarvis CK, Schaefer CP, Jacobs LM, Herndon JM, Hunn KC (2018). Acute pain alters P-glycoprotein-containing protein complexes in rat cerebral microvessels: Implications for P-glycoprotein trafficking. J Cereb Blood Flow Metab.

[43] Barakat S, Demeule M, Pilorget A, Regina A, Gingras D, Baggetto LG (2007). Modulation of p-glycoprotein function by caveolin-1 phosphorylation. J Neurochem.

[44] Noack A, Noack S, Hoffmann A, Maalouf K, Buettner M, Couraud PO (2014). Drug-induced trafficking of p-glycoprotein in human brain capillary endothelial cells as demonstrated by exposure to mitomycin C. PLoS One.

[45] Zhang F, Zhang H, Wang Z, Yu M, Tian R, Ji W (2014). P-glycoprotein associates with Anxa2 and promotes invasion in multidrug resistant breast cancer cells. Biochem Pharmacol.

[46] Darby RA, Unsworth A, Knapp S, Kerr ID, Callaghan R (2015). Overcoming ABCG2-mediated drug resistance with imidazo-[1,2-b]-pyridazine-based Pim1 kinase inhibitors. Cancer Chemother Pharmacol.

[47] Natarajan K, Bhullar J, Shukla S, Burcu M, Chen ZS, Ambudkar SV (2013). The Pim kinase inhibitor SGI-1776 decreases cell surface expression of P-glycoprotein (ABCB1) and breast cancer resistance protein (ABCG2) and drug transport by Pim-1-dependent and -independent mechanisms. Biochem Pharmacol.

[48] Xie Y, Burcu M, Linn DE, Qiu Y, Baer MR (2010). Pim-1 kinase protects P-glycoprotein from degradation and enables its glycosylation and cell surface expression. Mol Pharmacol.

[49] Xie Y, Xu K, Linn DE, Yang X, Guo Z, Shimelis H (2008). The 44-kDa Pim-1 kinase phosphorylates BCRP/ABCG2 and thereby promotes its multimerization and drug-resistant activity in human prostate cancer cells. J Biol Chem.

[50] Chen D, Zhang H, Lu P, Liu X, Cao H (2016). Synergy evaluation by a pathway–pathway interaction network: a new way to predict drug combination. Mol Biosyst.

[51] Kuhn M (1998). The microtubule depolymerizing drugs nocodazole and colchicine inhibit the uptake of Listeria monocytogenes by P388D1 macrophages. FEMS Microbiol Lett.

